# Are Melanocortin Receptors Present in Extant Protochordates?

**DOI:** 10.3390/biom14091120

**Published:** 2024-09-04

**Authors:** Ren-Lei Ji, Shan-Shan Jiang, Gunnar Kleinau, Patrick Scheerer, Ya-Xiong Tao

**Affiliations:** 1 Department of Anatomy, Physiology and Pharmacology, College of Veterinary Medicine, Auburn University, Auburn, AL 36849, USA; rlj0027@auburn.edu (R.-L.J.); szj0083@auburn.edu (S.-S.J.); 2 Charité–Universitätsmedizin Berlin, Corporate Member of Freie Universität Berlin and Humboldt-Universität zu Berlin, Institute of Medical Physics and Biophysics, Group Structural Biology of Cellular Signaling, D-10117 Berlin, Germany; gunnar.kleinau@charite.de (G.K.); patrick.scheerer@charite.de (P.S.)

**Keywords:** melanocortin receptor, chordates, cephalochordate, urochordata, evolution, constitutive activity, G protein-coupled receptor

## Abstract

Functional melanocortin receptor (MCR) genes have been identified in the genomes of early chordates, e.g., the cyclostomata. Whether they appear in the most ancient chordates such as cephalochordate and urochordata, however, remains unclear due to missing genetic data. Herein, we studied five putative (from NCBI database), sequence-based predicted MCR-like receptors from urochordata and cephalochordate, including *Styela clava*, *Ciona intestinalis*, *Branchiostoma floridae*, and *Branchiostoma belcheri*. The BLAST and phylogenetic analyses suggested a relationship between these specific receptors and vertebrate MCRs. However, several essential residues for MCR functions in vertebrates were missing in these putative chordata MCRs. To test receptor functionality, several experimental studies were conducted. Binding assays and functional analyses showed no specific binding and no ligand-induced cAMP or ERK1/2 signaling (with either endogenous α-MSH or synthetic ligands for MC4R), despite successfully expressing four receptors in HEK 293T cells. These four receptors showed high basal cAMP signaling, likely mediated by ligand-independent Gs coupling. In summary, our results suggest that the five predicted MCR-like receptors are, indeed, class A G protein-coupled receptors (GPCRs), which in four cases show high constitutive activity in the Gs-cAMP signaling pathway but are not MCR-like receptors in terms of ligand recognition of known MCR ligands. These receptors might be ancient G protein-coupled receptors with so far unidentified ligands.

## 1. Introduction

The melanocortin system consists of five melanocortin receptors (MCRs), endogenous agonists (melanocortin peptides), and two endogenous antagonists (agouti-related protein (AgRP) and agouti signaling protein (ASIP)) [1,2,3]. Melanocortin peptides are derived from the tissue-specific post-translational processing of the common precursor proopiomelanocortin (POMC), including α-, β-, γ-melanocyte-stimulating hormones (MSHs), and adrenocorticotropin (ACTH) [1]. The multiple biological functions of these peptides are exerted by MCRs, members of rhodopsin-like Family A G protein-coupled receptors (GPCRs), including modulation of dermal pigmentation, immune response, adrenocortical steroidogenesis, lipolysis, stress, cardiovascular function, energy homeostasis, appetite regulation, exocrine secretion, and timing of sexual maturation [2,3,4,5,6,7]. MCR activation is primarily coupled to the stimulatory heterotrimeric G protein (Gs), as well as to phosphorylates extracellular signal-regulated kinase 1 and 2 (ERK1/2). The signaling and pharmacological characteristics of the MCRs are additionally influenced by a cluster of transmembrane proteins known as melanocortin-2 receptor accessory proteins (MRAPs) [8,9,10,11,12].

The phylum Chordata includes three sub-phyla: cephalochordate, urochordata, and vertebrates. The most ancient known versions of MCRs, MCa and MCb, have been identified in lampreys so far [13,14]. Tetrapod species possess five MCRs. However, the number of receptors varies in teleost fish. The zebrafish, for example, has six *mcr* genes, including two copies of *mc5r*, while the pufferfish has only four *mcr* genes, lacking the *mc3r*. The loss of *mc3r* has been observed in many teleosts [15]. Based on the chromosome localization and gene structure of MCRs, these receptors can be classified into two branches resulting from the ancestral duplication of MCRs during the first round of whole-genome duplication (1R). The MCa branch subsequently underwent duplication events, giving rise to MC1R and the ancestral MC2R/MC5R. The MCb branch underwent an initial duplication event, which produced two distinct gene copies and then underwent further divergence, leading to the formation of MC3R and MC4R [16]. The ancestral form of MC2R/MC5R was then duplicated through a local event, resulting in the emergence of MC2R and MC5R. MCR genes appear to be a chordate gene family, as orthologues have been identified and documented in the genomes of various chordate species, including hagfish, lamprey, cartilaginous fish (sharks and rays), teleosts (bony fish), and tetrapods (amphibians, reptiles, birds, and mammals). However, these genes might be absent in the genomes of echinoderms, cephalocordates, urochordates, as well as protostomes [17].

The co-evolution of melanocortin peptides and MCRs is substantiated by the presence of the *pomc* gene and the ancestral *mcr* gene during the early radiation of chordates [18,19,20]. To date, both MCR-related and POMC-related genes have been identified in sea lamprey [13,14]. POMC is an ancient gene, most likely derived from an ancestral opioid-coding gene following the 1R chordate genome duplication event [21]. POMC belongs to the opioid/orphanin gene family, including proenkephalin (PENK), proorphanin (PNOC), and prodinorphyn (PDYN) [22]. In tetrapod species, POMC comprises the N-terminal pro-c-MSH, the central ACTH, and the C-terminal b-lipotropin. Each domain contains one MSH peptide characterized by a core binding motif sequence of *HFRW*. POMC was first discovered in sea lamprey, the ancient vertebrate, with two different POMC orthologues (proopiomelanotropin (POM) and proopiocortin (POC)) [23]. POM only exhibits an MSH-core and opioid sequences, whereas POC encodes ACTH and β-endorphin [23]. In teleosts, γ-MSH is absent [24,25]. α-MSH is very much conserved in vertebrates from cyclostomata to mammals [24,25]. To date, the evolutionary origin of POMC remains uncertain. It is unclear whether POMC emerged before the lamprey/gnathostome division. However, genes related to opioids/orphanins and MCRs have not been found in the genomes of cephalochordates (amphioxus or lancelets) or in urochordata (tunicates) [26].

Cephalochordates and urochordates represent the most ancient chordates. Although they lack an internal bony or cartilaginous skeleton, the ancestors of modern cephalochordates and urochordates gave rise to vertebrates, as these progenitors share many features with vertebrates and are considered their origins. Recently, some genomes of amphioxus and tunicates have been published [27,28,29], improving our understanding of chordate evolution and the origin of vertebrates, and providing an opportunity to investigate the evolution of GPCRs in vertebrates.

Interestingly, four putative, *mc4r*-like receptor genes, and one putative *mc1r*-like receptor gene from urochordata and cephalochordate, including *Styela clava* (sc), *Ciona intestinalis* (ci), *Branchiostoma floridae* (bf), and *Branchiostoma belcheri* (bb), were recently predicted in these genomes. To test whether these receptors are, indeed, MCR-like (hereafter called Mcrs or Mcr-like to separate them from the vertebrate MCRs), we tested them pharmacologically in cell-based assays and explored putative ligand-binding and -signaling capacities, accompanied by a detailed sequence comparison and a search for POMC encoding genes.

## 2. Materials and Methods

### 2.1. Gene Cloning, Amino Acid Sequence Alignments, and Phylogenetic Analyses

Initially, we searched the NCBI database using keywords like “MC4R”, “MCR”, or “melanocortin receptor”, along with terms like “amphioxus”, “lancelets”, or “tunicates”. Subsequently, we retrieved the *mc1r* sequence for *Ciona intestinalis* (GenBank: XM_002120933.5), the *mc4r* sequence for *Styela clava* (GenBank: XM_039403826.1), the *mc4r* sequence for *Branchiostoma floridae* (GenBank: XM_035814192.1), as well as the *mc4r*1 (GenBank: XM_019766663.1) and *mc4r*2 (GenBank: XM_019765569.1) sequences for *Branchiostoma belcheri* from the NCBI database (https://www.ncbi.nlm.nih.gov/, accessed on 5 February 2022) using this systematic search method.

All sequence alignments of these and various other receptors were conducted using Clustal X2 with the parameters: BLOSUM matrix; gap opening 10; gap extension 0.2, iteration of each alignment step [30]. Several manual justifications were done on the received alignment to correct, e.g., gaps in the sequences of transmembrane helix (TMH) regions.

The resulting alignment (FASTA format) was used for phylogenetic analyses by maximum likelihood methods with 50 bootstraps in the Jones–Taylor–Thornton model within Molecular Evolutionary Genetics Analysis (Mega 11 software) [31]. The phylogenetic tree was visualized with iTOL (https://itol.embl.de/, Version 6.9.1, accessed on 8 June 2024).

### 2.2. Ligands and Plasmids

[Nle^4^, D-Phe^7^]-α-MSH (NDP-α-MSH) was purchased from Vivitide (Louisville, KY, USA). Human α-MSH was obtained from Pi Proteomics (Huntsville, AL, USA). [^125^I]-NDP-α-MSH and [^125^I]-cAMP was iodinated using the chloramine T method [32,33]. N-terminal myc-tagged receptors (ciMc1r, scMc4r, bfMc4r, bbMc4r-1 and bbMc4r-2) were commercially synthesized and subcloned into pcDNA3.1 by GenScript (Piscataway, NJ, USA). The N-terminal myc-tagged human MC4R (hMC4R) and N-terminal myc-tagged dog MC1R (dMC1R) subcloned into pcDNA3.1 vector were generated as previously described [34,35]. The N-terminal 3xHA-tagged human MC1R (hMC1R) subcloned into pcDNA3.1 vector was purchased from cDNA Resource Center (https://www.cdna.org/).

### 2.3. Cell Culture and Transfection

Human embryonic kidney (HEK) 293T cells were purchased from ATCC (Manassas, VA, USA) and cultured in a 5% CO_2_-humidified incubator at 37 °C with the medium contained Dulbecco’s Modified Eagle’s medium, 10% newborn calf serum, 50 mg/mL gentamicin, 0.25 mg/mL amphotericin B, 100 mg/mL streptomycin, 100 IU/mL penicillin, and 10 mM HEPES [34]. At 70% confluency, cells were transfected with plasmids at 0.25 μg/μL using the calcium phosphate precipitation method [36].

### 2.4. Flow Cytometry Assay

Flow cytometry (Accuri Cytometers, Ann Arbor, MI, USA) was used to investigate the expression of receptors in transiently transfected HEK293T cells, as described previously [37]. The fluorescence of cells transfected with empty vector (pcDNA3.1) was set as background staining.

### 2.5. Ligand-Binding Assays

A binding assay was performed, as described previously [34,38]. Briefly, 48 h after transfection, cells were washed with warm DMEM/bovine serum albumin (DMEM/BSA) and then incubated with DMEM/BSA containing ~80,000 cpm [^125^I]-NDP-α-MSH with a buffer or 10^−5^ M of unlabeled α-MSH for 1 h [34]. Cells were washed with cold Hank’s balanced salt, lysed with 0.5 M NaOH, and collected using cotton swabs. The radioactivity was determined by a Gamma counter (Cobra II Auto-Gamma, Packard Bioscience, Frankfurt, Germany).

### 2.6. Intracellular cAMP Assays

Intracellular cAMP levels were determined by radioimmunoassay (RIA), as described previously [32,34]. To explore the constitutive activity of cAMP signaling, cells were transfected with increasing concentrations of plasmids (0, 0.007, 0.015, 0.030, 0.060, 0.125, and 0.250 μg/μL). A total of 48 h after transfection, cells preincubated with DMEM/BSA containing 0.5 mM isobutylmethylxanthine (Sigma-Aldrich, St. Louis, MO, USA) for 30 min were further incubated for 1 h. To examine the effects of hMC4R inverse agonists on the cAMP levels of putative Mc4rs, cells expressing receptors were treated with 10 nM AgRP, 1 μM MCL0020, 1 μM Ipsen 5i, or 1 μM ML00253764 for 1 h.

### 2.7. ERK1/2 Phosphorylation Assay

The pERK1/2 levels were detected by immunoblotting, as described previously [39,40,41,42]. A total of 24 h after transfection, cells were starved in DMEM/BSA for 24 h, and then stimulated with buffer or different endogenous ligands and drugs (1 μM α-MSH, 10 nM AgRP, 1 μM Ipsen 5i, 1 μM ML00253764, or 1 μM MCL0020) for 5 min [33,39]. Mouse anti-β-tubulin antibody (Developmental Studies Hybridoma Bank, University of Iowa, Iowa City, IA, USA) and rabbit anti-pERK1/2 antibody (Cell Signaling, Beverly, MA, USA) were used in this study. The membranes were quantified by ImageJ 1.44 (NIH, Bethesda, MD, USA).

### 2.8. Statistical Analysis

All data were represented as mean ± S.E.M. GraphPad Prism 8.3 software (GraphPad, San Diego, CA, USA) was used to calculate the parameters of ligand binding, cAMP signaling, and flow cytometry assay. The significances were determined by the Student’s *t*-test between two groups. The one-way ANOVA test was used to analyze the significant differences among multiple groups.

## 3. Results

### 3.1. Nucleotide and Deduced Amino Acid Sequences of Mcr-like Genes in Lancelets and Tunicates

Five *mcr-like* genes (*Branchiostoma floridae mc4r*, *Branchiostoma belcheri mc4r1*, *Branchiostoma belcheri mc4r2*, *Styela clava mc4r*, and *Ciona intestinalis mc1r*) were identified in lancelets and tunicates through the NCBI database by searching specific keywords.

The predicted *Styela clava mc4r* (scMc4r like) had a 1170 bp open reading frame (ORF), encoding a putative protein of 389 amino acids, *Branchiostoma floridae mc4r* (bfMc4r like) had a 966 bp ORF, encoding a putative protein of 321 amino acids, *Branchiostoma belcheri mc4r1* (bbMc4r like1) had a 1221 bp ORF, encoding a putative protein of 406 amino acids, *Branchiostoma belcheri mc4r2* (bbMc4r like2) had a 1158 bp ORF, encoding a putative protein of 385 amino acids, and *Ciona intestinalis mc1r* (ciMc1r like) had a 1017 bp ORF, encoding a putative protein of 338 amino acids.

To determine if these receptors were, indeed, part of the MCR family, we conducted a sequence-based BLAST search on NCBI (via blastp model) with the five extracted receptor sequences. The results showed that these receptors, except for bbMc4r1, had moderate alignment scores between 73 and 106 (numerical values used to quantify the similarity between sequences, such as protein sequences) and high query coverage percentages (73–93%) (indication of how much of the query sequence is covered by the aligned segments in the subject sequence), along with low E-values to MCRs (Supplementary Table S1). A sequence-similarity analysis based on the comparison of entire proteins of MCR subtypes and predicted Mcr-like receptors revealed similarities between 41 and 50% (Supplementary Table S2), which can be seen as low conservation.

**Figure 1:**
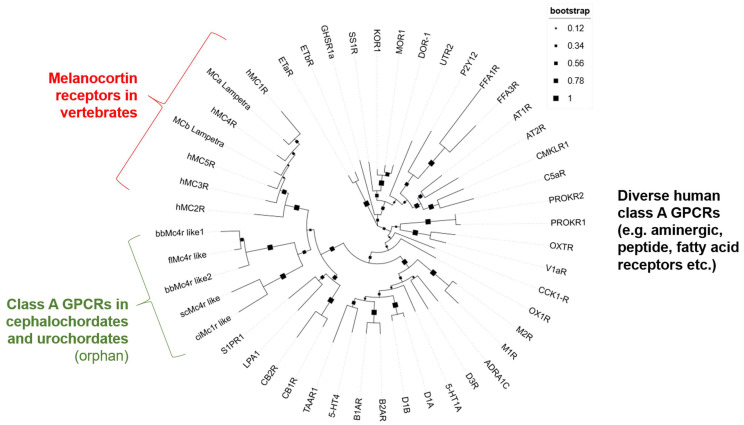
Phylogenetic tree of human MCRs, putative Mcrs, and diverse other human class A GPCRs. This phylogenetic tree is based on the alignment of transmembrane helices according to the determined MC4R structure (PDB ID: 7PIV) [43]. The compared class A GPCRs included all human MCR subtypes, MCRs of lamprey, but also several other and diverse receptors of these groups to ensure reasonable analysis parameters. Finally, the receptors SP1R, LPA1, and cannabinoid receptors (CB1R and CB2R) were included based on predicted higher similarities of the entire sequences. All protein IDs used are listed in Supplementary Table S3.

To further estimate the relationships between predicted Mcrs and vertebrate MCRs, and because our sequence-based BLAST-search with the, e.g., bbMc4r like1, also revealed higher sequence identities to other class A GPCRs than the MCRs, such as the lysophosphatidic acid receptor 1 (LPA1)-like (54% sequence identity), we generated a phylogenetic tree based on a sequence alignment of the transmembrane helices of MCRs, Mcrs, and diverse members of class A GPCRs. This analysis demonstrated that the predicted Mcrs form a subgroup clustered beside the MCRs of vertebrates close to a GPCR sub-branch constituted by the cannabinoid (CB) receptors, LPARs, and sphingosin-1-phosphat-receptors (S1PRs) (Figure 1). Other class A GPCRs such as aminergic or fatty acid receptors are phylogenetically distant.

**Figure 2:**
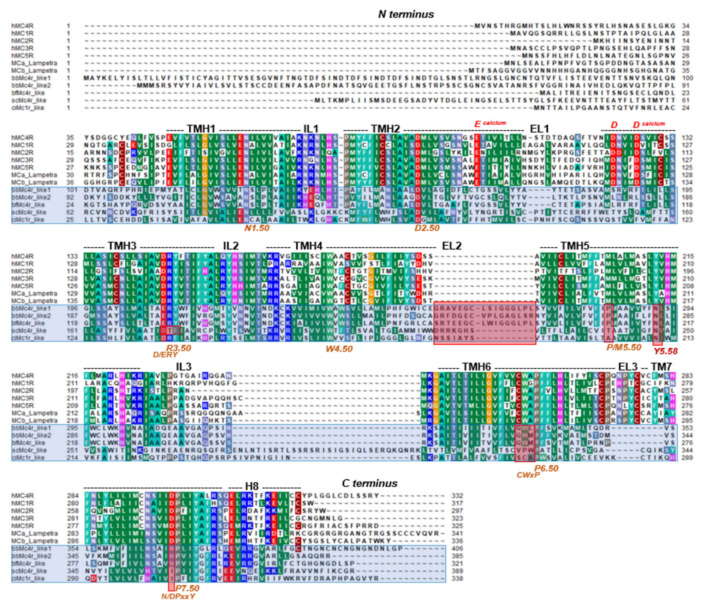
Sequence comparison between MCRs (human, lamprey) and predicted Mcr-like receptors of cephalochordates and urochordates. This alignment shows the comparison of full-length receptor sequences with annotated structural specificities as the dimension of helices derived from the solved MC4R structure (PDB ID: 7PIV) [43]. Moreover, conserved amino acid motifs typical for class A GPCRs are highlighted, including the Ballesteros and Weinstein numbers. A calcium ion is an essential co-binding factor for MCR ligands, and is justified between negatively charged amino acids in TMH2 and TMH3 (*EDD* binding motif), which are highlighted in red above the sequences. These residues are missing in the Mcr-like receptors (blue translucent boxes), and other deviations from typical MCR sequences can be observed (red translucent boxes). For example, the EL2 is much longer and the *CWxP* motif in TMH6 that is essential for receptor activation is different. The alignment was visualized with the software BioEdit version 7.2.5 [46]. Specific background colors of conserved amino acid positions reflect specific properties of the amino acid side chains or the type of amino acid: black—proline; blue—positively charged; cyan/green—aromatic and hydrophobic; green—hydrophobic; red—negatively charged; gray—hydrophilic; dark red—cysteines; and magenta—histidine. (TMH—transmembrane helix; EL—extracellular loop; IL—intracellular loop; H8—helix 8).

In the next step, we explored in depth the sequences of extracted Mcr-like proteins compared to human and lamprey MCRs. MCRs are characterized by specific amino acids (MCR sequence fingerprint) responsible for calcium ion and ligand binding, as well as amino acid residues and motifs that are common in class A GPCRs [43,44]. With this comparison, we estimated how likely identical functionalities of Mcr-like proteins are compared to known vertebrate MCRs (Figure 2).

First, the compared receptors obviously shared classical GPCR features, such as seven TMHs (transmembrane helices) connected by extracellular (EL) and intracellular loops (IL), and an extracellular N terminus and an intracellular C-terminus. Several highly conserved residues such as N^1.50^, D^2.50^, R^3.50^, W^4.50^, P^6.50,^ and P^7.50^ (the superscript numbers are based on the numbering scheme of Ballesteros and Weinstein [45]), as well as conserved amino acid motifs (*D/ERY*, and *D/NPxxY*), exist in these receptors, indicating these receptors as members of class A GPCRs (Figure 2).

**Figure 3:**
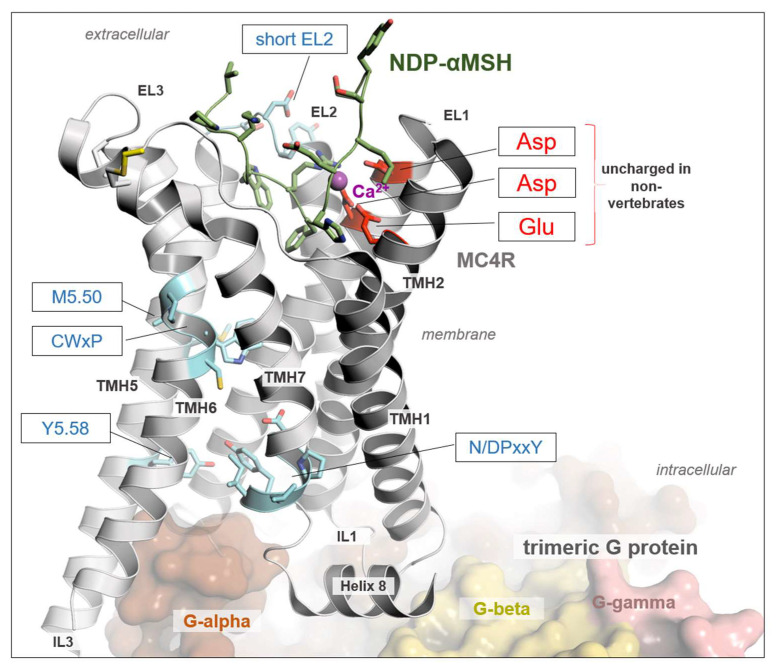
Structure of MC4R in complex with G protein and a peptide agonist NDP-α-MSH. The determined structure of hMC4R in complex with binding partners in an active state (PDB ID: 7PIV) [43] reveals detailed insights into ligand, G protein, and calcium binding. A calcium ion is bound between negatively charged amino acid residues in TMH2 and TMH3, which are not present in the Mcr-like receptors investigated here (Figure 2). The calcium ion is an obligate peptide-ligand binding co-factor, as evidenced by several MCR complex structures. Moreover, amino acid side chains are also highlighted by sticks and are associated with the signaling regulation and transduction of MCRs, as are the *CWx*P, the *N/DPxxY* motif, and most class A GPCRs. They are also different in predicted Mcr-like receptors. Finally, a short EL2, as observed in all evidenced MCRs of vertebrates, is longer in Mcr-like receptors, which most likely would hamper the binding and justification of POMC-derived ligands in a competitive manner. This structural representation was generated using the PyMOL Molecular Graphics System Version 2.5.5 (Schrödinger, LLC, New York, NY, USA).

However, crucial residues for MCR ligands are absent in these putative Mcrs (Figure 2). For instance, the negatively charged E100 (TMH2), and D122 or D126 (TMH3) of hMC4R, or their corresponding residues in all hMCR orthologous [43,47,48,49,50,51], are obligated to bind the essential co-ligand binding factor calcium, but they were not present in these putative Mcrs. Additionally, vertebrate MCRs typically have a short EL2, whereas the putative Mcrs exhibited an extended and long EL2, which likely interferes with the binding of hormone ligands derived from POMC in a mode known from the diverse MCR 3D structures available. These ligands bind centrally in the receptor in an extracellular crevice (Figure 3), which is not accessible for ligands in other GPCRs with longer ELs. Furthermore, significant differences between MCRs and Mcrs in amino acid compositions known to be of relevance for signaling and signal regulation can be observed in TMH6 (*CWxP* motif), in TMH7 (*N/DPxxY* motif), but also at the position 5.58, which is usually a tyrosine (Tyr) in class A GPCRs but substituted in Mcrs by an asparagine (Asn). Of note, at this position in the thyroid-stimulating hormone receptor (TSHR), a Tyr-Asn substitution is known to induce higher basal signaling activity [52].

To summarize, while the overall sequence similarity may suggest relationships between these receptors in cephalochordates and urochordates with vertebrate MCRs, a detailed sequence analysis of residues known to be essential for MCR functions (MCR-sequence fingerprint) reveals strong differences. These differences (e.g., the absence of a calcium ion binding site, which is mandatory for the orientation and stability of various MCR ligands) [43,49,50,51,53], suggest that the predicted Mcr-like receptors might not have comparable ligands or similar functions in cephalochordates and urochordates. To test this hypothesis, several further experimental studies were conducted.

### 3.2. POMC in Cephalochordates and Urochordates?

We examined the genomes of these species for other genes of the melanocortin system. Initially, we conducted searches within the NCBI database using both abbreviated and full gene names related to the melanocortin system, such as POMC, POM, POC, AgRP, and MRAP. Additionally, we performed BLAST searches using gene and protein sequences of these genes from closely related species like hagfish and lamprey against the genomic data of amphioxus and urochordates. Furthermore, to enhance sensitivity, we conducted BLAST searches for the POMC gene using the minimal MSH peptide coding sequences (His-Phe-Arg-Trp). However, we did not identify any genes coding for *Pomc*, *Mrap*, *Agrp*, or *Asip* in the genomes of these species.

### 3.3. Expression and Specific Binding of Putative Mc4rs-like Receptors

**Figure 4:**
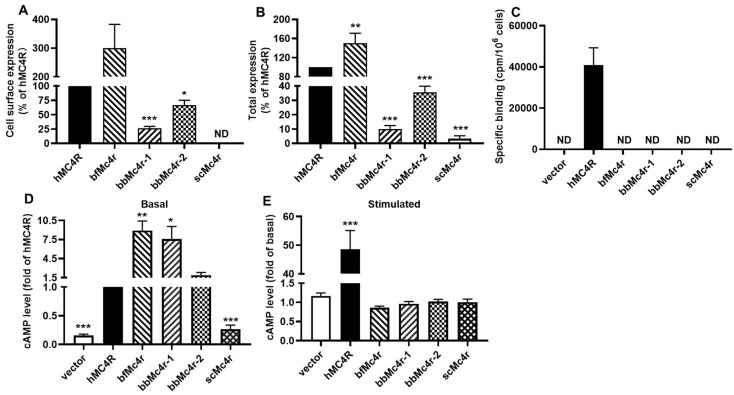
Expression, specific binding, and signaling of hMC4R and the putative Mc4rs. (**A**,**B**) cell surface and total expression of the receptors. HEK293T cells were transfected with hMC4R and putative Mc4rs. Receptor expression was determined by flow cytometry. Fluorescence of cells transfected with empty vector (pcDNA3.1) was set as background staining. The expression of receptors was calculated as the percentage of cells transfected with hMC4R (set as 100%). (**C**) Specific binding of hMC4R and putative Mc4rs. (**D**) Basal cAMP signaling of hMC4R and putative Mc4rs. (**E**) α-MSH-induced signaling of hMC4R and putative Mc4rs. All experiments were repeated at least three independent times. * Indicates significantly different from hMC4R (*p* < 0.05), ** indicates significantly different from hMC4R (*p* < 0.01), and *** indicates significantly different from hMC4R (*p* < 0.001). ND, could not be determined.

We initially investigated the cell surface and total expression of putative Mc4rs in HEK293T cells using flow cytometry (Figure 4A,B). The results indicated that, except for scMc4r, three putative Mc4rs (bfMc4r, bbMc4r-1, and bbMc4r-2) were successfully expressed in transiently transfected HEK293T cells, demonstrating substantial levels of cell surface and total expression (Figure 4A,B). However, the binding assay results revealed that no specific binding to NDP-α-MSH was observed in HEK293T cells expressing these four receptors (Figure 4C).

### 3.4. Gs-cAMP Signaling of Putative Mc4rs

We further examined the basal and α-MSH-induced Gs-cAMP signaling of these putative Mc4rs. Three putative Mc4rs (bfMc4r, bbMc4r-1, and bbMc4r-2) exhibited significantly higher basal Gs-cAMP signaling compared to hMC4R, with levels 8.9, 7.6, and 1.9 times higher, respectively, whereas scMc4r displayed similar basal cAMP levels as the empty vector control (Figure 4D). Furthermore, none of the four receptors demonstrated α-MSH-stimulated Gs-cAMP signaling (Figure 4E).

**Figure 5:**
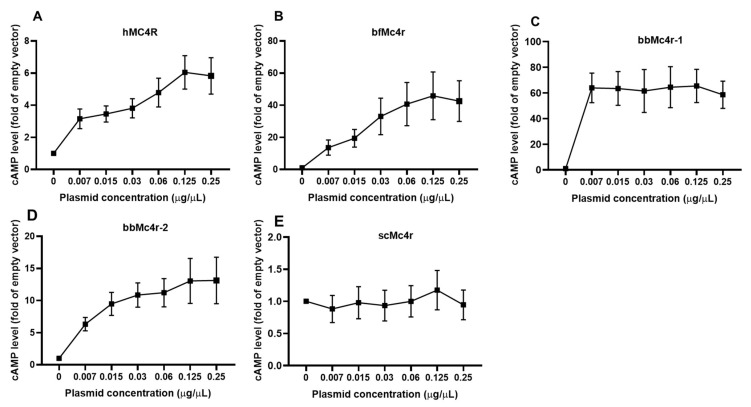
Constitutive activities of hMC4R and the putative Mc4rs in cAMP signaling. (**A**) hMC4R; (**B**) bfMc4r; (**C**) bbMc4r-1; (**D**) bbMc4r-2; and (**E**) scMc4r. HEK293T cells were transfected with increasing concentrations of MC4R plasmids. Cells transfected with empty vector pcDNA3.1 were considered as a control group (set at 1.0). cAMP levels were measured by RIA. The curve was created with data from three or five independent experiments.

To investigate in detail the potential constitutive activity of these receptors, we transfected cells with increasing concentrations of putative Mc4r plasmids. The results demonstrated that, as the plasmid concentrations increased, cAMP levels were elevated in cells expressing hMC4R, bfMc4r, and bbMc4r-2 (Figure 5). Notably, even with a very low amount of bbMc4r-1 plasmid (0.007 μg/μL) transfection, a high level of cAMP was observed (Figure 5C). However, despite increasing concentrations of transfected scMc4r, there was no induction of cAMP generation (Figure 5E). These findings indicate that three receptors (bfMc4r, bbMc4r-1, and bbMc4r-2) were potentially coupled to the Gs protein, exhibiting a high level of constitutive activity in Gs-cAMP signaling.

### 3.5. Effects of Five Compounds on Both Gs-cAMP and ERK1/2 Signaling of Putative Mc4rs

**Figure 6:**
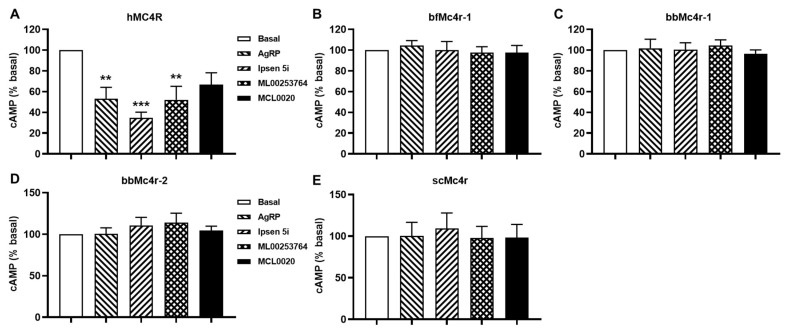
Effects of four ligands on the basal activities of MC4Rs. (**A**) hMC4R; (**B**) bfMc4r; (**C**) bbMc4r-1; (**D**) bbMc4r-2; and (**E**) scMc4r. All experiments were repeated at least three independent times. ** indicates significantly different from basal (*p* < 0.01), and *** indicates significantly different from basal (*p* < 0.001).

The elevated basal activity observed in both wild-type (WT) and constitutively active mutant hMC4Rs could be attenuated by various inverse agonists, including AgRP (83–132), Ipsen 5i, ML00253764, and MCL0020 [40,54,55]. To investigate the effects of these inverse agonists on the basal activities of the putative Mc4rs, cells transfected with these receptors were treated with 10 nM AgRP, 1 μM Ipsen 5i, 1 μM MCL0020, or 1 μM ML00253764, respectively. However, the results indicated that three ligands (AgRP, Ipsen 5i, and ML00253764) decreased the basal cAMP levels of hMC4R, whereas none of these hMC4R inverse agonists was able to modulate the cAMP signaling of any of the tested receptors (Figure 6).

**Figure 7:**
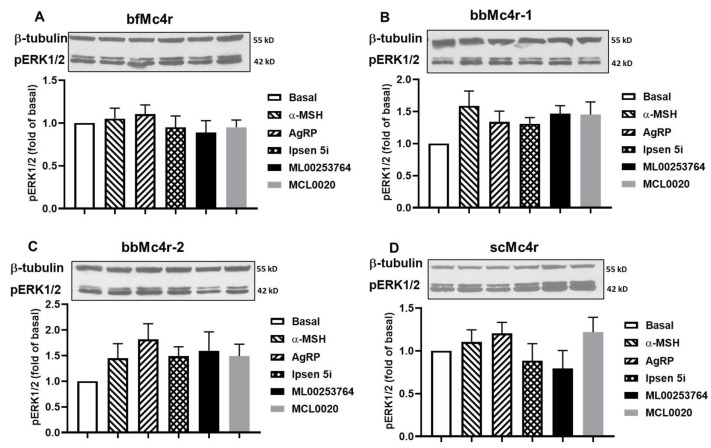
ERK1/2 signaling properties of the putative Mc4rs. Ligand-stimulated pERK1/2 levels in cells expressing bfMc4r (**A**), bbMc4r-1 (**B**), bbMc4r-2 (**C**), and scMc4r (**D**). All experiments were repeated at least three independent times. Original western blots can be found in Supplementary Materials.

In addition to Gs-cAMP signaling, vertebrate MCRs also activate the ERK1/2 signaling pathway [56,57,58,59]. Hence, we further investigated whether these receptors could activate ERK1/2 signaling. Cells expressing these receptors were treated with buffer, 1 μM α-MSH, 10 nM AgRP, 1 μM Ipsen 5i, 1 μM MCL0020, or 1 μM ML00253764, respectively. The results showed that none of the five compounds tested could alter the ERK1/2 signaling pathway of these four putative Mc4rs (Figure 7).

### 3.6. Pharmacology of Putative Mc1r

**Figure 8:**
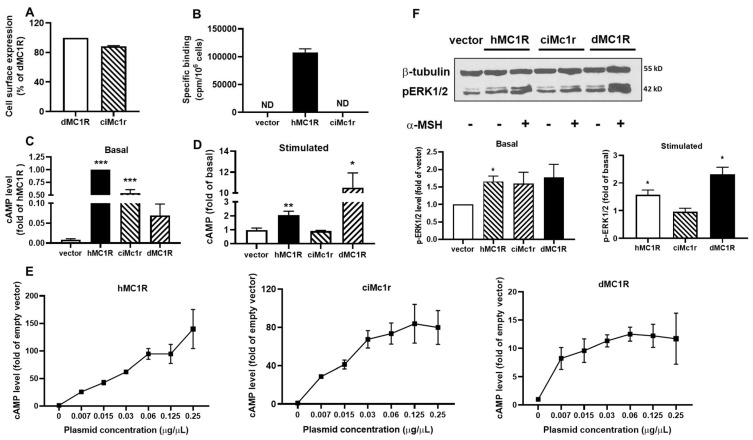
Pharmacology of the putative Mc1r. (**A**) Cell surface expression of dMC1R and the putative Mc1r; (**B**) specific binding of hMC1R and ciMc1r; (**C**) basal cAMP signaling of hMC1R, dMC1R, and the putative Mc1r; (**D**) α-MSH-induced signaling of hMC1R, dMC1R, and the putative Mc1r; (**E**) constitutive activities of hMC1R, dMC1R, and the putative Mc1r in cAMP signaling; and (**F**) ERK1/2 signaling properties of hMC1R, dMC1R, and the putative Mc1r. Original western blots can be found in Supplementary Materials. All experiments were repeated at least three independent times. * Indicates significantly different from empty vector (*p* < 0.05); ** indicates significantly different from empty vector (*p* < 0.01), and *** indicates significantly different from empty vector (*p* < 0.001). ND, could not be determined.

We also discovered a *mc1r*-like gene from *Ciona intestinalis* and conducted further pharmacological studies on the receptor, ciMc1r. The results indicated that ciMc1r had similar cell surface expression to dMC1R, suggesting that ciMc1r was expressed in HEK293T cells (Figure 8A). Similar to the putative Mc4rs, ciMc1r did not demonstrate specific binding to NDP-α-MSH either (Figure 8B). In terms of signaling, both hMC1R and ciMc1r demonstrated high basal cAMP signaling, while dMC1R displayed lower basal cAMP signaling (Figure 8C). α-MSH was capable of stimulating cAMP generation in hMC1R and dMC1R, but it failed to induce cAMP signaling in ciMc1r (Figure 8D). Transfection of low concentrations of hMC1R and dMC1R, as well as ciMc1r plasmids, resulted in a significant increase in cAMP levels (Figure 8E), indicating constitutive activation of ciMc1r. Furthermore, ciMc1r showed no basal or α-MSH-induced ERK1/2 signaling (Figure 8F).

## 4. Discussion

Cephalochordates and urochordates, the earliest known chordates, are believed to have given rise to vertebrates, with cephalochordates (amphioxus) at the base of chordates and urochordates as the closest living relatives of vertebrates [60]. The study of these organisms holds significant importance in our understanding of evolution [28]. MCRs play crucial roles in various physiological functions. Previous studies have failed to identify *mcr*-related genes in cephalochordates and urochordates, leading to the hypothesis that MCRs originate from the time of the lamprey’s first appearance [13,14]. In this study, we investigated five genes from cephalochordates and urochordates provided by the NCBI database that were annotated as “Mcr-like receptors”.

We examined the receptors bfMc4r, bbMc4r1, bbMc4r2, scMc4r, and ciMc4r using BLAST to ascertain their association with the MCR family. Our analysis revealed notable alignment scores and significant resemblance to MCRs for all receptors as expected (Supplementary Table S1). In addition, we conducted a phylogenetic tree analysis, which showed that the predicted Mcrs form a distinct cluster along with class A GPCRs, especially close to MCRs, cannabinoid receptors, LPA receptors, and S1P receptors (Figure 1), which provides at least some evidence for the classification of these receptors as part of the class A GPCRs. In addition, a detailed sequence analysis showed that these receptors did not present several MCR-typical amino acids essential for ligand binding and signal transduction (Figure 2 and Figure 3).

These insights are consistent with our experimental findings. The results from binding assays and functional studies demonstrated a lack of specific binding and no ligand-induced cAMP or ERK1/2 signaling (with either endogenous α-MSH or synthetic ligands for MC4R), despite the successful expression of four receptors in the HEK 293T cells (Figure 4, Figure 5, Figure 6, Figure 7 and Figure 8). Thus, based on our data, we propose that these receptors might not be MCRs.

One explanation might be that an ancestral *mcr* gene was present in these lineages, while the POMC gene may never have evolved in them. In hagfish (jawless vertebrates, organisms older than lamprey), an *mcr* gene (MCc, GenBank: DQ213061.1) was discovered, showing high similarities to MCRs of sea lamprey. However, genomic analysis of hagfish does not reveal the presence of POMC-related genes, including POMC, POM, and POC. In contrast, lamprey possesses two receptors (MCa and MCb) and three POMC-related genes (*POMC*, *POM*, and *POC*), supporting the notion that *MCR* genes may have arisen earlier than *POMC* genes in jawless vertebrates [13,14].

Alternatively, these ancestral Mcrs might have mutated further to acquire new functions [61,62]. This scenario is intricate and requires specific evidence. However, examples exist where ancestral GPCR genes underwent loss-of-function mutations and later evolved new functions [63,64]. For instance, the opsin gene family, responsible for encoding photoreceptor proteins in vertebrates, illustrates this phenomenon. Some ancestral opsin genes accumulated mutations, rendering them non-functional for photoreception. However, some of these non-functional opsin genes evolved new functions, such as regulating circadian rhythms or influencing skin pigmentation [63,64]. Similar phenomena are observed in the *TAS2R38* gene and the *TAS2R* gene family, which encode bitter taste receptors in mammals [31,65]. These examples highlight the dynamic nature of GPCR evolution and the potential for functional diversification following loss-of-function mutations.

Finally, our data demonstrate at least that, except for scMc4r, the investigated GPCRs from cephalochordates and urochordates were functional receptors that were constitutively active and preferentially coupled with the Gs protein. Constitutive activity, signaling in the absence of an agonist, is a significant pharmacological characteristic of many GPCRs [66]. However, the receptors investigated herein are still orphans and awaiting the identification of their ligands and their physiological functions accordingly, which might be different from known MCR-related topics.

## 5. Conclusions

In summary, these putative Mcrs did not exhibit typical vertebrate MCR characteristics when tested with common endogenous and synthetic MCR ligands, or by comparing MCR-specific sequence fingerprints, but a certain overall sequence similarity was present. Three of the putative Mc4rs and one Mc1r exhibited functionality, showing high constitutive activity and a preference for coupling with the Gs protein. This suggests that these receptors may represent ancient class A GPCRs with so far unidentified ligands.

## Data Availability

The raw data supporting the conclusions of this article will be made available by the authors upon request, without undue reservation.

## Supplementary Materials

The following supporting information can be downloaded at: https://www.mdpi.com/article/10.3390/biom14091120/s1, Table S1: BLAST score table; Table S2: Amino acid sequence similarities between supposed MCR like receptors and MCR subtypes as well as other class A GPCRs supposed to have high similarity scores; Table S3: GPCR protein IDs.
