# Supplementary material for: Are Melanocortin Receptors Present in Extant Protochordates?
Source: Biomolecules. 2024 Sep 4;14(9):1120. doi: 10.3390/biom14091120 (PMC11430673; doi:10.3390/biom14091120)
Supplement: Supplementary file 1 [file biomolecules-14-01120-s001.zip › biomolecules-3166109-supplementary.pdf]

Are melanocortin receptors present in extant protochordates?

Ren-Lei Ji [1], Shan-Shan Jiang [1], Gunnar Kleinau [2], Patrick Scheerer [2], and Ya-Xiong Tao [1*]

[1] Department of Anatomy, Physiology and Pharmacology, College of Veterinary Medicine, Auburn University, Auburn, AL 36849, United Statesrlj0027@auburn.edu; szj0083@auburn.edu

[2] Charité – Universitätsmedizin Berlin, corporate member of Freie Universität Berlin, Humboldt-Universität zu Berlin, Institute of Medical Physics and Biophysics, Group Structural Biology of Cellular Signaling, D-10117, Berlin, Germany; gunnar.kleinau@charite.de; patrick.scheerer@charite.de

* Correspondence: taoyaxi@auburn.edu; Tel.: +1 3348445396 (Y.X.T)

Suppl. Table S1: BLAST score table

	Species	Max score	Query coverage	E-value	Identities	Accession
		bfMc4r				
melanocortin-4 receptor	*Synchiropus splendidus*	104	93%	2.00E-21	27.9	XP_053716097.1
melanocortin-4 receptor-like	*Echeneis naucrates*	104	94%	2.00E-21	27.66	XP_029384825.1
melanocortin-4 receptor	*Paralichthys olivaceus*	100	91%	4.00E-20	26.67	BAK61811.1
melanocortin-4 receptor	*Lates calcarifer*	100	92%	8.00E-20	27.02	XP_018534777.1
		bbMc4r1				
cannabinoid receptor 2	*Ahaetulla prasina*	108	71%	3.00E-22	28.34	XP_058051859.1
G protein-coupled receptor 3	*Erpetoichthys calabaricus*	105	78%	4.00E-21	28.26	XP_028675508.1
G protein-coupled receptor 186	*Polypterus senegalus*	103	78%	2.00E-20	27.64	XP_039596396.1
G-protein coupled receptor 6	*Xenopus laevis*	96.7	68%	5.00E-18	28.52	XP_018120269.1
Sphingosine 1-phosphate receptor	*Osmerus mordax*	96.7	75%	6.00E-18	27.62	ACO09498.1
		bbMc4r2				
melanocortin-4 receptor	*Paralichthys olivaceus*	106	77%	1.00E-21	29.61	BAK61811.1
melanocortin-4 receptor	*Boleophthalmus pectinirostris*	105	73%	3.00E-21	30.42	XP_020784806.1
melanocortin-4 receptor	*Lates calcarifer*	101	75%	8.00E-20	29.78	XP_018534777.1
melanocortin-4 receptor	*Scophthalmus maximus*	99.8	78%	3.00E-19	29.27	AWP21094.1
		scMc4r				
melanocortin-5 receptor	*Mesocricetus auratus*	75.9	89%	8.00E-11	23.08	NP_001274540.1
melanocortin-4 receptor-like	*Salmo salar*	73.9	79%	3.00E-10	23.6	XP_014013065.1
melanocortin-5 receptor	*Phodopus roborovskii*	73.6	79%	3.00E-10	22.73	CAH7033270.1
melanocortin-4 receptor	*Oncorhynchus mykiss*	72.8	79%	9.00E-10	23.29	XP_021476310.1
melanocortin-5 receptor	*Mus musculus*	69.7	78%	7.00E-09	22.33	NP_038624.3
		ciMc1r				
melanocortin-4 receptor-like	*Acipenser ruthenus*	83.2	93%	2.00E-13	27.02	XP_058874749.1
melanocortin-5 receptor-like	*Megalops cyprinoides*	79.7	93%	2.00E-12	27.38	XP_036411389.1
melanocortin-4 receptor	*Polyodon spathula*	79	85%	4.00E-12	27.46	XP_041102527.1
melanocortin 5a receptor	*Cololabis saira*	73.2	85%	3.00E-10	26.49	XP_061573884.1

adrenocorticotropic hormone receptor-like	*Myxocyprinus asiaticus*	73.2	85%	3.00E-10	25.57	XP_051574493.1

BLAST score table were calculated using blastp via online tool (https://blast.ncbi.nlm.nih.gov/Blast.cgi?PROGRAM=blastp&PAGE_TYPE=BlastSearch&LINK_LOC=blasthome)

Suppl. Table S2: Amino acid sequence similarities between supposed MCR like receptors and MCR subtypes as well as other class A GPCRs supposed to have high similarity scores.

	**bfMc4r**	**bbMc4r-1**	**bbMc4r-2**	**scMc4r**	**ciMc1r**	**MCa**	**MCb**	**hMC1R**	**hMC2R**	**hMC3R**	**hMC4R**	**hMC5R**	**hS1PR1**	**hLPAR1**	**hCB2R**
**bfMc4r**	100	67	63	42	45	49	48	49	48	49	49	49	41	46	46
**bbMc4r-1**		100	65	45	40	40	41	41	38	41	44	43	42	40	38
**bbMc4r-2**			100	46	39	44	43	44	41	41	44	42	40	44	40
**scMc4r**				100	52	43	45	44	42	43	47	46	43	33	41
**ciMc1r**					100	46	47	46	46	47	49	50	44	45	42
**MCa**						100	67	62	57	68	66	69	46	47	44
**MCb**							100	63	59	75	76	74	47	47	41
**hMC1R**								100	57	62	64	63	42	45	42
**hMC2R**									100	62	62	63	44	41	42
**hMC3R**										100	73	77	46	48	44
**hMC4R**											100	76	46	47	41
**hMC5R**												100	48	46	46
**hS1PR1**													100	58	44
**hLPAR1**														100	45
**hCB2R**															100

MCa (ABB36647.1), MCb (ABB36648.1), bfMc4r (XP_035670085.1), bbMc4r-1 (XP_019622222.1), bbMc4r-2 (XP_019621128.1), scMc4r (XP_039259760.1), ciMc1r (XP_002120969.1), hS1PR1 (NP_001391.2), hLPAR1 (AAP84359.1), hMC1R (NP_002377.4), hMC2R (NP_000520.1), hMC3R (AAO72726.1), hMC4R (AAO92061.1), hMC5R (NP_005904.1), and hCB2R (AAO92299.1). Protein similarities were calculated using online tool (https://en.vectorbuilder.com/tool/sequence-alignment.html).

Suppl. Table S3 GPCR protein IDs

GPCR name	Protein ID
hETaR	AAP32294.1
hETbR	AY275463.1
hGHSR1a	AAR07907.1
hSS1R	AAR25625.1
hKOR1	AAM21070.1
hMOR1	AAS00462.1
hDOR1	NP_000902.3
hUTR2	NP_061822.1
hP2Y12	AAN01280.1
hFFA1R	NP_005294.1
hFFA3R	NP_005295.1
hAT1R	NP_000676.1
hAT2R	NP_000677.2
hCMKLR1	AAR90850.1
hC5aR	NP_001727.2
hPROKR2	NP_658986.1
hPROKR1	NP_620414.1
hOXTR	AAQ91333.1
hV1aR	AAP84363.1
hCCK1R	AAP84362.1
hOX1R	AAL47214.1
hM2R	AAM18939.1
hM1R	AAM18938.1
hADRA1C	NP_000671.2
hD3R	NP_000787.2
h5-HT1A	AAM21125.1
hD1A	NP_000785.1
hD1B	NP_000789.1
Hβ_2AR	NP_000015.1
hβ_1AR	NP_000675.1

h5-HT4	NP_000861.1
hTAAR1	AAO22154.1
hCB1R	NP_057167.2
hCB2R	AAO92299.1
hLPA1	AAP84359.1
hS1PR1	NP_001391.2
hMC1R	NP_002377.4
hMC2R	NP_000520.1
hMC3R	AAO72726.1
hMC4R	AAO92061.1
hMC5R	NP_005904.1
lamprey MCa	ABB36647.1
lamprey MCb	ABB36648.1
bfMc4r	XP_035670085.1
bbMc4r1	XP_019622222.1
bbMc4r2	XP_019621128.1
scMc4r	XP_039259760.1
ciMc1r	XP_002120969.1

Figure S7A

HEK293T cells expressing empty vector or **bfMc4r**

Cells treated with 1 μM α-MSH, 10 nM AgRP, 1 μM Ipsen 5i, 1 μM ML00253764 or 1 μM MCL0020 for 5 min

Primary antibody:

Rabbit anti-pERK1/2 antibody (Catalog # 4370, Cell Signaling (1:1000); mouse anti-β-tubulin antibody (Developmental Studies Hybridoma Bank, University of Iowa, Iowa City, IA) (1:5000)

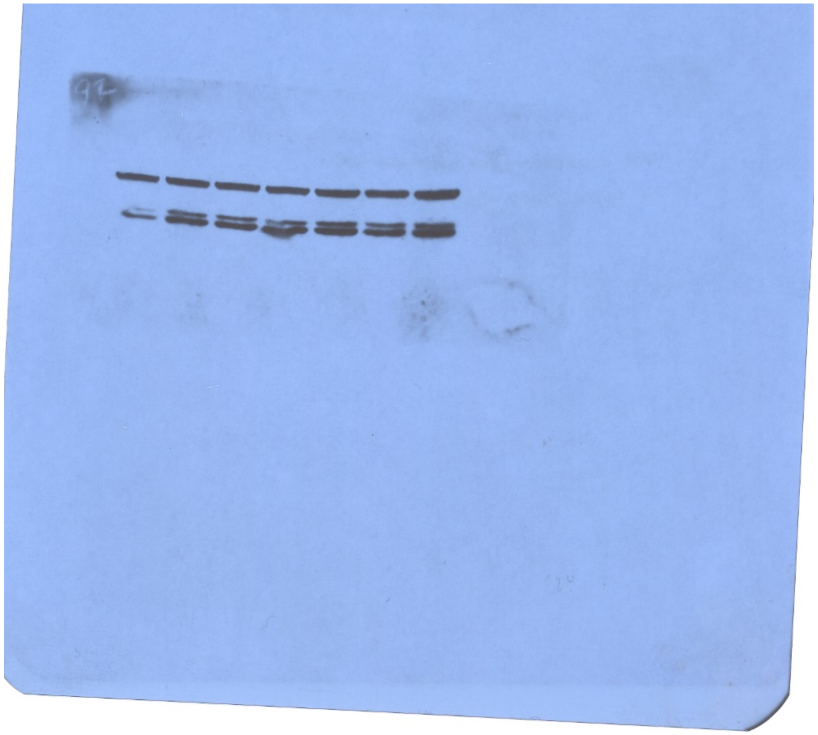

Figure S7B

HEK293T cells expressing empty vector or **bbMc4r-1**

Cells treated with 1 µM α-MSH, 10 nM AgRP, 1 µM Ipsen 5i, 1 µM ML00253764 or 1 µM MCL0020 for 5 min

Primary antibody:

Rabbit anti-pERK1/2 antibody (Catalog # 4370, Cell Signaling (1:1000);
mouse anti-β-tubulin antibody (Developmental Studies Hybridoma Bank, University of Iowa, Iowa City, IA) (1:5000)

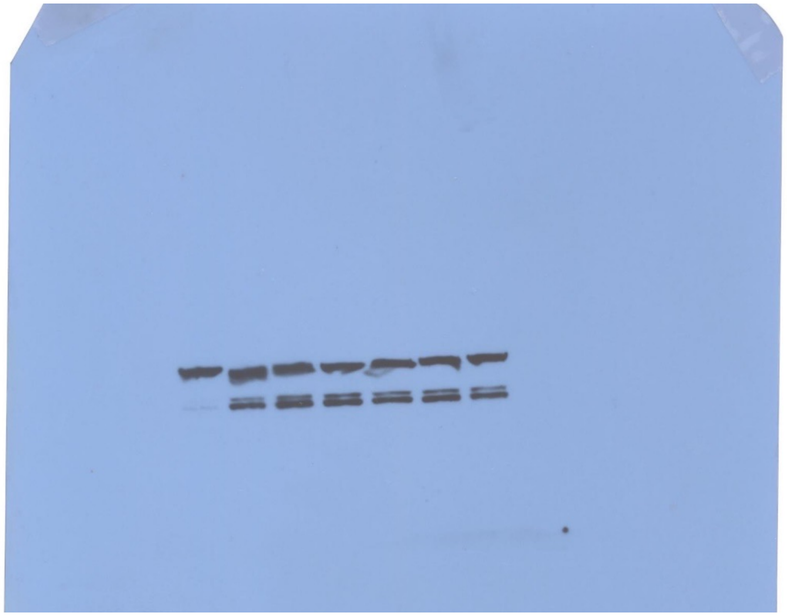

Figure S7C

HEK293T cells expressing empty vector or **bbMc4r-2**

Cells treated with 1 μM α-MSH, 10 nM AgRP, 1 μM Ipsen 5i, 1 μM ML00253764 or 1 μM MCL0020 for 5 min

Primary antibody:

Rabbit anti-pERK1/2 antibody (Catalog # 4370, Cell Signaling (1:1000);
mouse anti-β-tubulin antibody (Developmental Studies Hybridoma Bank, University of Iowa, Iowa City, IA) (1:5000)

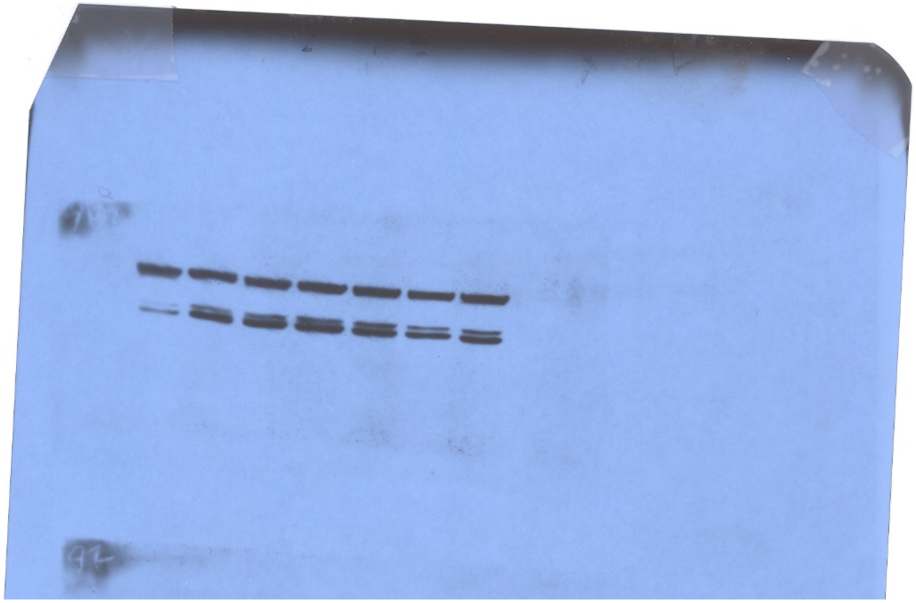

Figure S7D

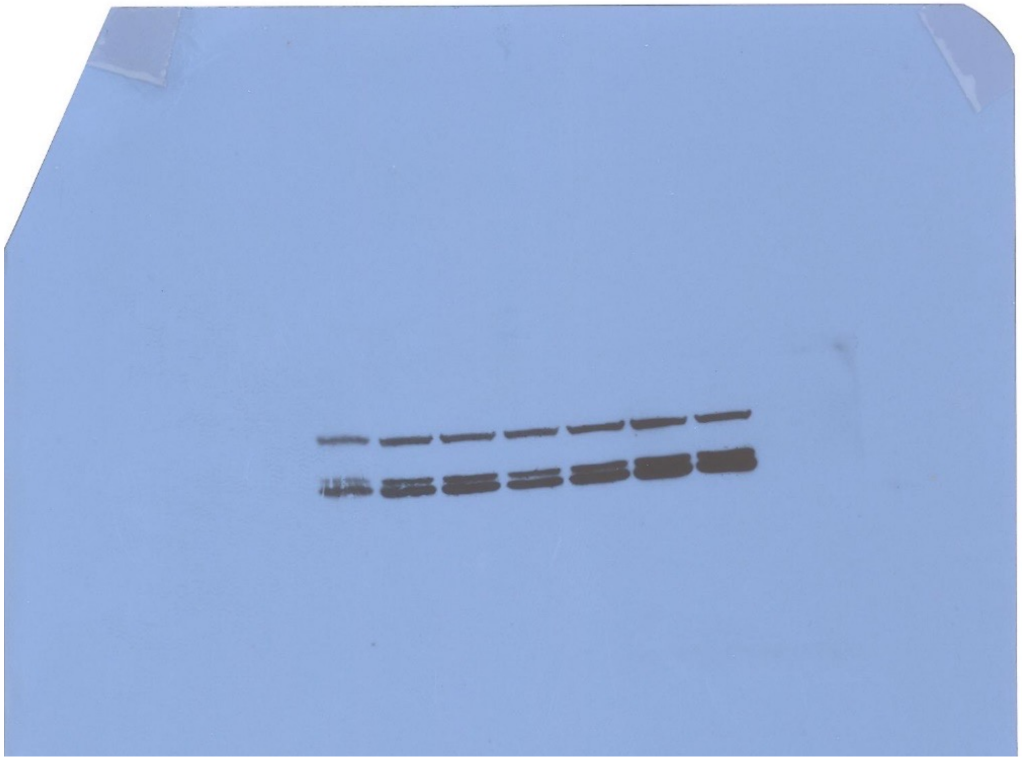

HEK293T cells expressing empty vector or **scMc4r**

Cells treated with 1 μM α-MSH, 10 nM AgRP, 1 μM Ipsen 5i, 1 μM ML00253764 or 1 μM MCL0020 for 5 min

Primary antibody:

Rabbit anti-pERK1/2 antibody (Catalog # 4370, Cell Signaling (1:1000); mouse anti-β-tubulin antibody (Developmental Studies Hybridoma Bank, University of Iowa, Iowa City, IA) (1:5000)

Figure S8F

HEK293T cells expressing empty vector or **hMC1R, ciMc1r or dMC1R**

Cells treated with buffer or 1 μM α-MSH for 5 min

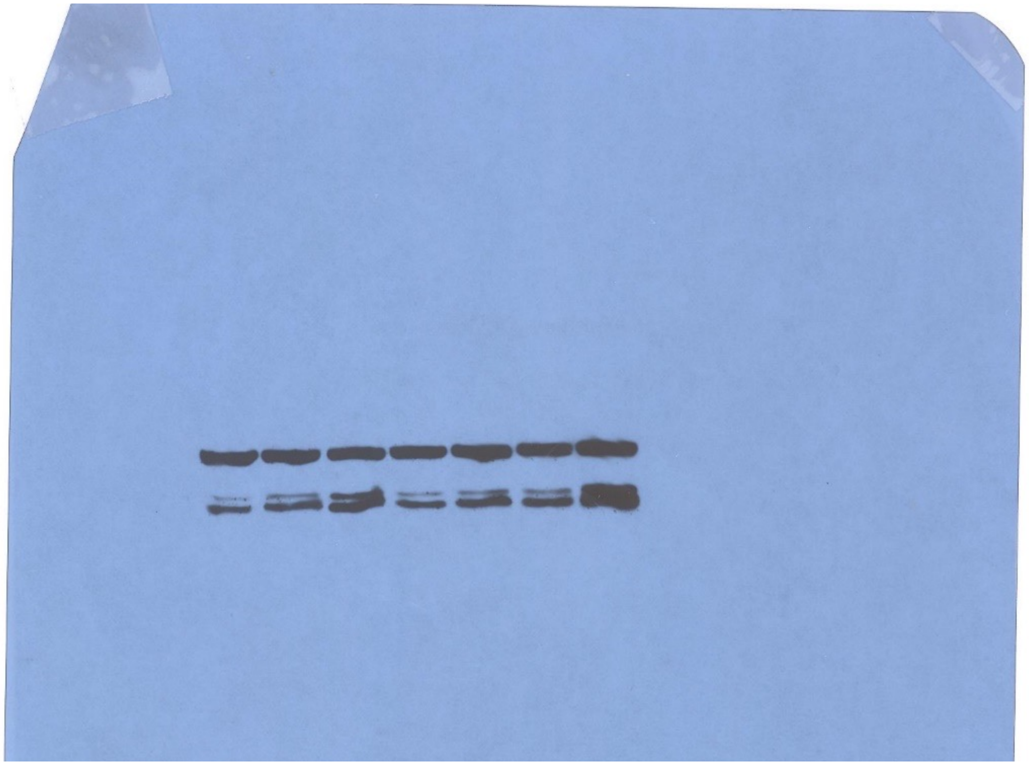

Primary antibody:

Rabbit anti-pERK1/2 antibody (Catalog # 4370, Cell Signaling (1:1000);
mouse anti-β-tubulin antibody (Developmental Studies Hybridoma Bank, University of Iowa, Iowa City, IA) (1:5000)
